# Supplementary material for: Spatial exosome analysis using cellulose nanofiber sheets reveals the location heterogeneity of extracellular vesicles
Source: Nat Commun. 2023 Nov 8;14:6915. doi: 10.1038/s41467-023-42593-9 (PMC10632339; doi:10.1038/s41467-023-42593-9)
Supplement: Supplementary file 1 — Supplementary information [file 41467_2023_42593_MOESM1_ESM.pdf]

Inventory of Supplementary Information

Supplementary Figures:

Supplementary Figure 1-15

Supplementary Table:

Supplementary Figures

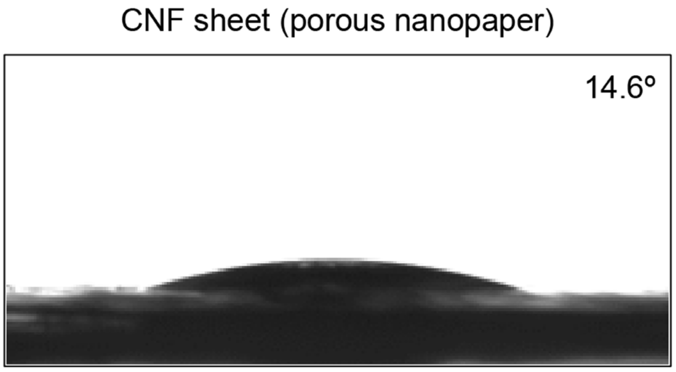

Supplementary Figure 1. Optical images and contact angles of water droplets on porous nanopaper after 10 s. Droplet volume: 1 µL. The porous nanopaper was prepared by drying in the presence of *tert*-butyl alcohol (*t*-BuOH) with a low surface tension. The porous nanopaper, i.e., the EV sheet, showed a low water-contact angle indicating that the tailored porous nanostructures within the EV sheet enhance its water absorbency.

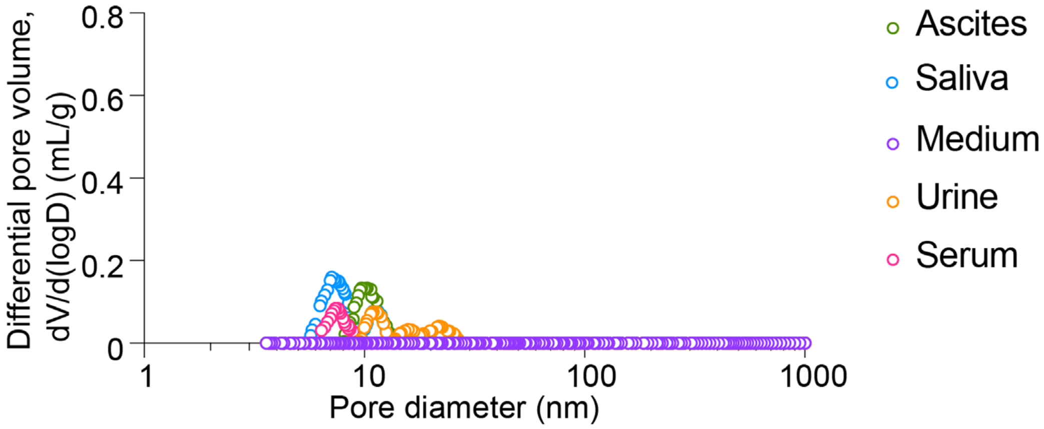

Supplementary Figure 2. Pore size distribution in CNF sheets determined using mercury intrusion method after supplying fluid (10 µL of medium, urine, serum, ascitic fluid, and saliva) and 7 days of drying. Each experiment was repeated at least three times.

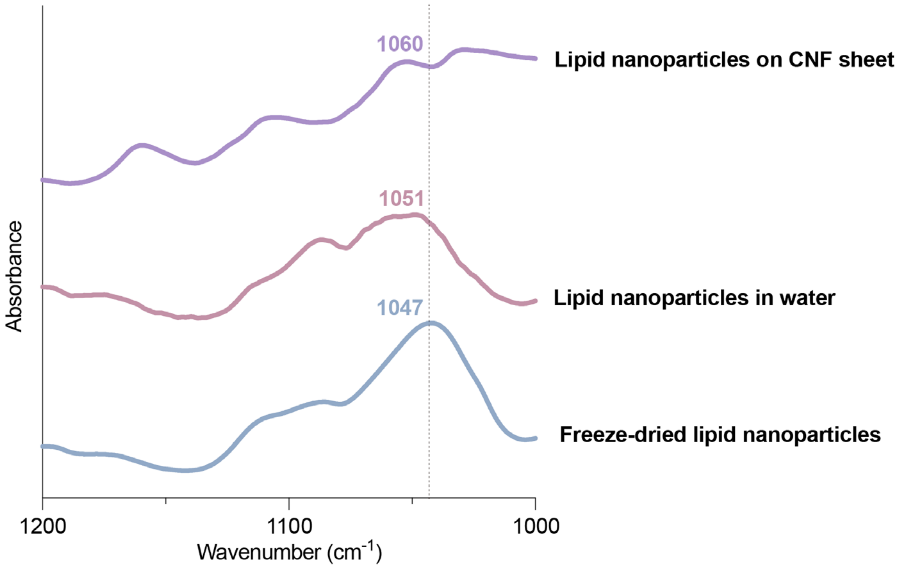

Supplementary Fig. 3. Infrared (IR) spectra of freeze-dried lipid nanoparticles, lipid nanoparticles dispersed in ultrapure water, and lipid nanoparticles on the CNF sheet. The vertical dashed line indicates the wavenumber of 1047 cm^{-1}.

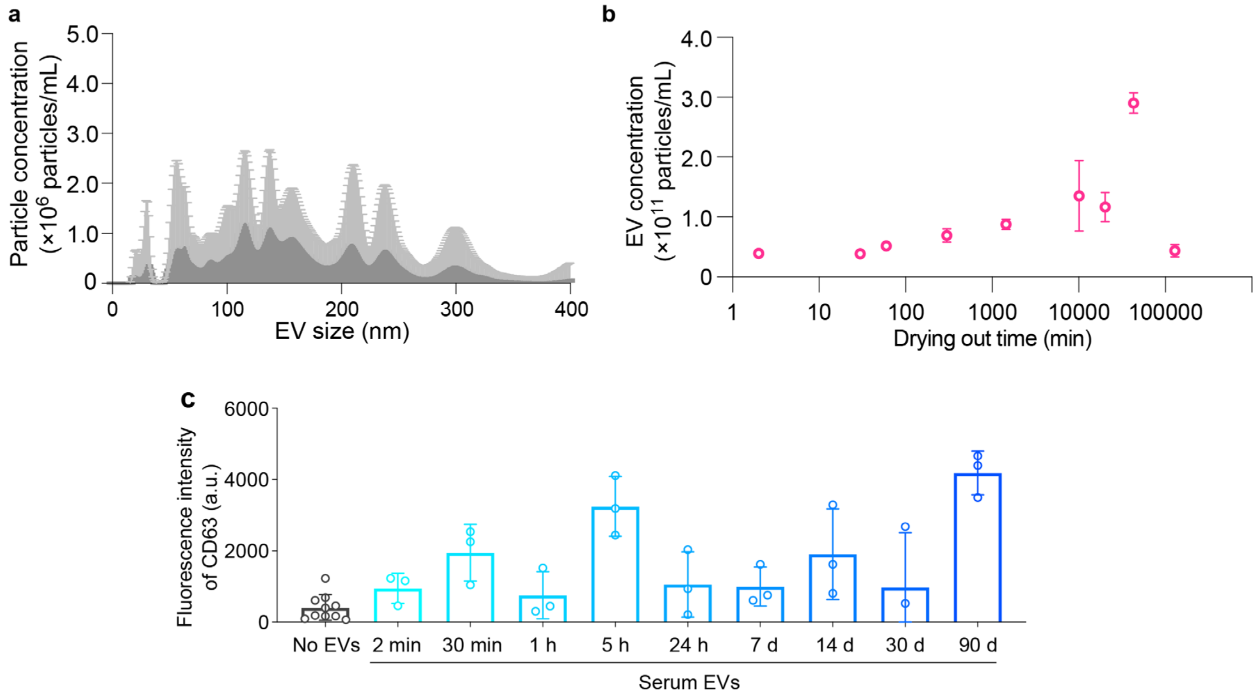

Supplementary Figure 4. (a) Size distribution of contaminants recovered from 10 µL of 0.22 µm-filtered PBS using EV sheets. (b) EV concentration at storage time in EV sheet. The storage times were 2 min, 30 min, 1 h, 5 h, 24 h, 7 d, 14 d, 30 d, and 90 d. (c) CD63 detection of EVs after various storage times in EV sheets. The storage times were 2 min, 30 min, 1 h, 5 h, 24 h, 7 d, 14 d, 30 d, and 90 d. All data are presented as mean values with SD. Each experiment was repeated at least three times.

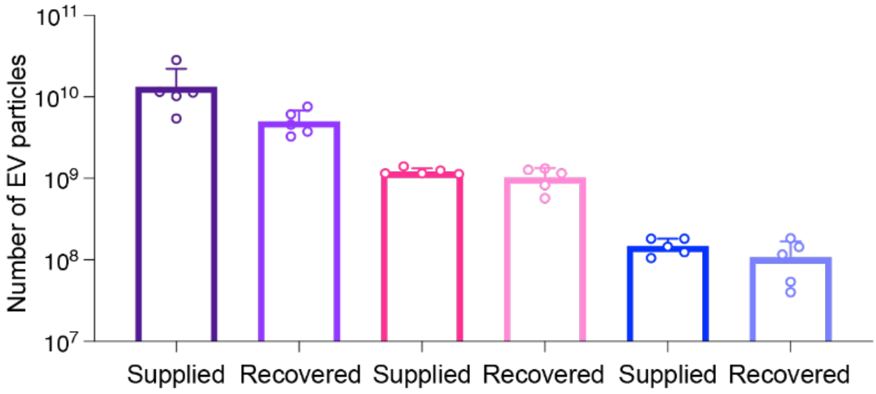

Supplementary Figure 5. EV recovery relative to EV supply at various EV concentrations. We observed that when lower amounts of EVs were supplied (e.g., 10^8 and 10^9 particles), the recovery was comparable to the input. However, at higher EV concentrations (e.g., 10^{10} particles), the recovery reached a saturation point at around 5×10^9 particles. Data are presented as mean values with SD. Each experiment was repeated at least three times.

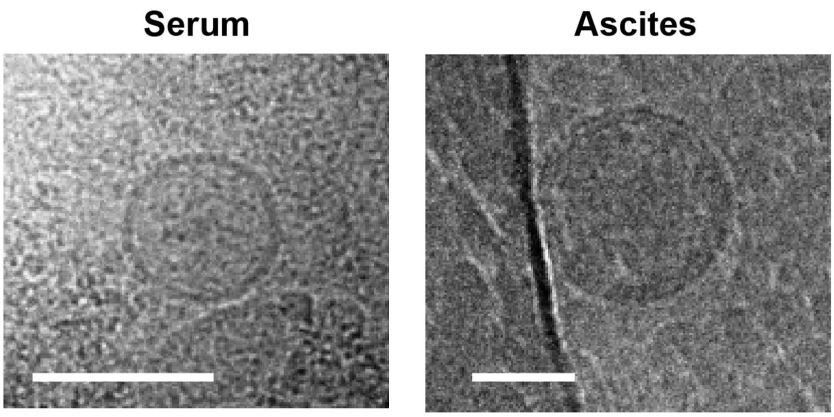

Supplementary Figure 6. cryo-EM images of serum and ascites EVs obtained using serial centrifugation. Scale bars: 100 nm.

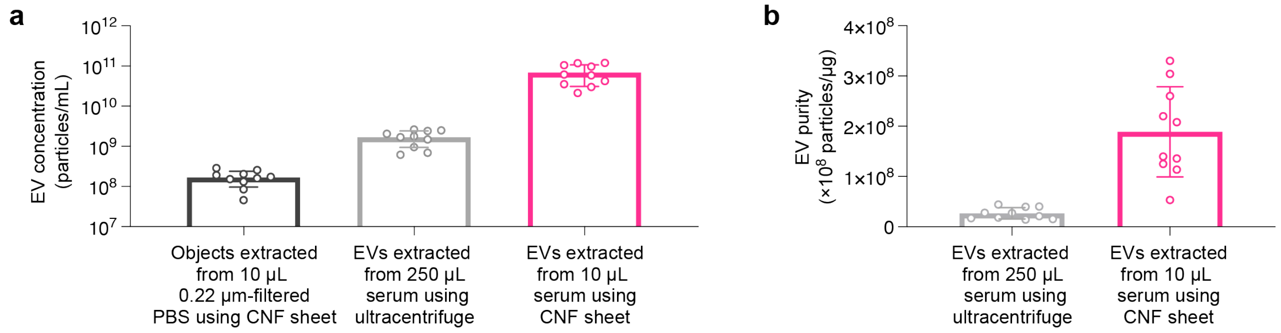

Supplementary Figure 7. Comparison of concentration and purity of EVs extracted using the various methods. (a) Concentration of EVs extracted from 10 μL of 0.22 μm-filtered PBS using the CNF sheet, from 250 μL of serum using ultracentrifugation, and from 10 μL of serum using the CNF sheet. (b) EV purity was calculated as the EV concentration (particles/mL) divided by the protein concentration (μg/mL). EV concentration was measured by NTA and protein concentration was measured by Qubit. All data are presented as mean values with SD. Each experiment was repeated at least three times.

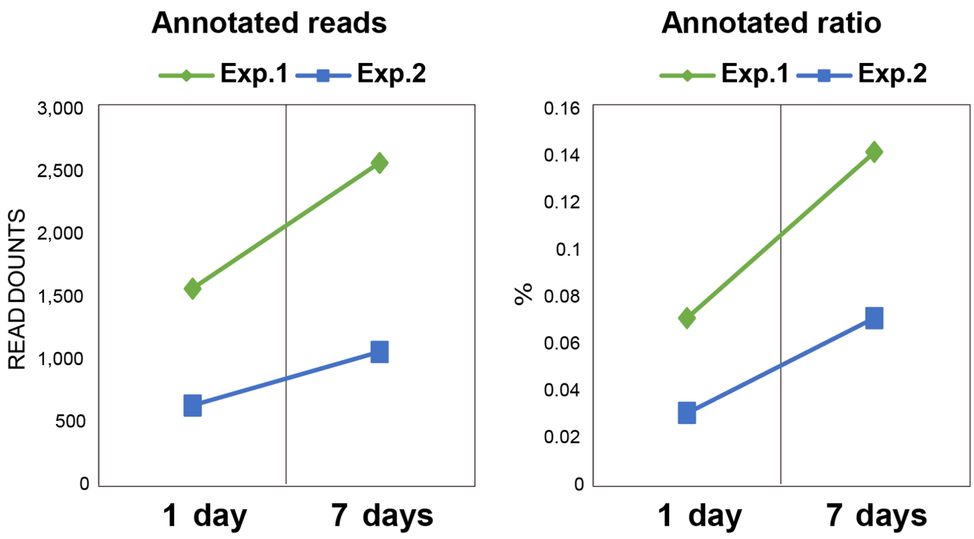

Supplementary Figure 8. Annotated read counts and ratios as miRNAs by small RNA sequencing for EV RNA from EV sheet attachment methods. The data from two independent sequencing analyses from the same sample are shown. Each experiment was repeated at least three times.

a

Serial No.	EV sheet	Diseases	Histopathological subtypes	Stage	Cytology	Age, years
Patient 1	Ex vivo	Ovarian carcinoma	High grade serous	III	Positive	71
Patient 2	Ex vivo	Ovarian carcinoma	Endometrial	III	Positive	61
Patient 3	In vivo	Ovarian carcinoma	Endometrial	I	Negative	43
Patient 4	In vivo	Ovarian borderline tumor	Mucinous	I	Negative	33
Patient 5	In vivo	Ovarian carcinoma	Clear cell	I	Negative	57
Patient 6	In vivo	Ovarian carcinoma	Mucinous	III	Positive	53

b

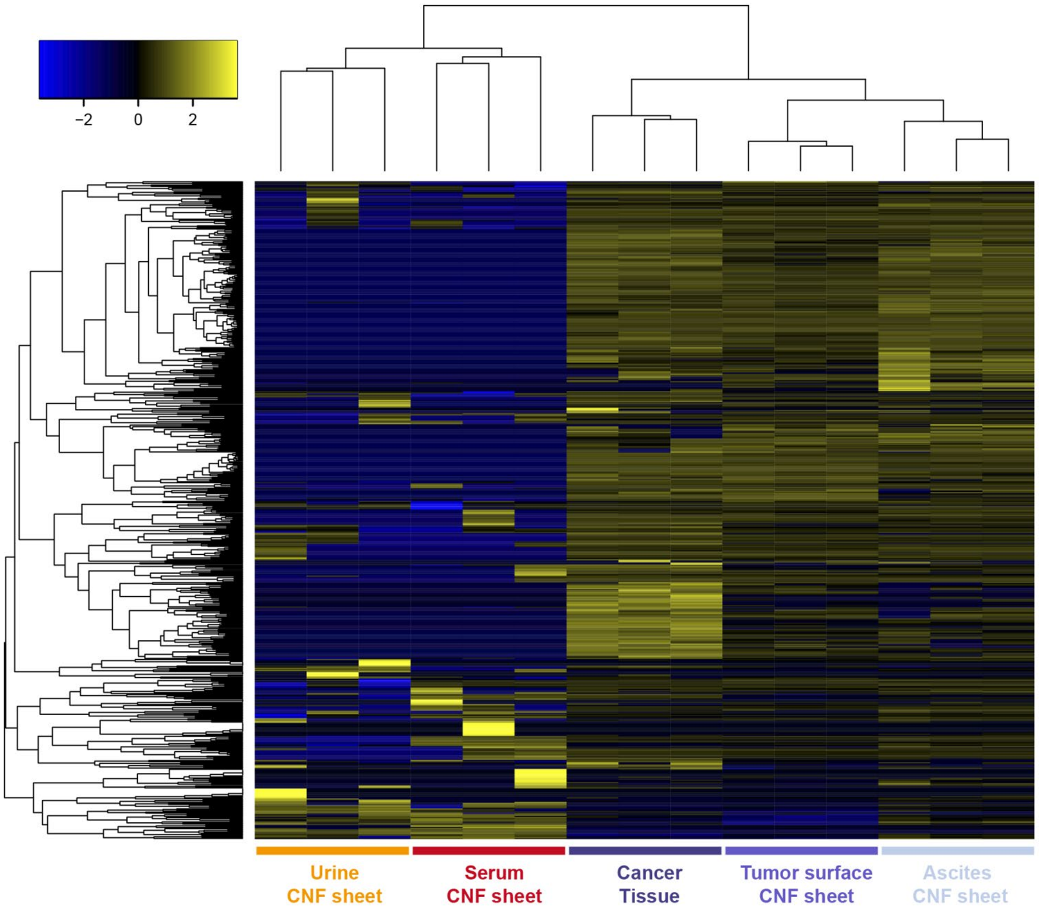

Supplementary Figure 9. (a) Characteristics of patients whose EVs were collected by EV sheet attachment methods. (b) A heatmap showing 599 expressed miRNAs from EV-sheet EVs based on miRNA sequencing from patient 1. Except for cancer tissue samples, all EVs in body fluids were captured by EV sheets.

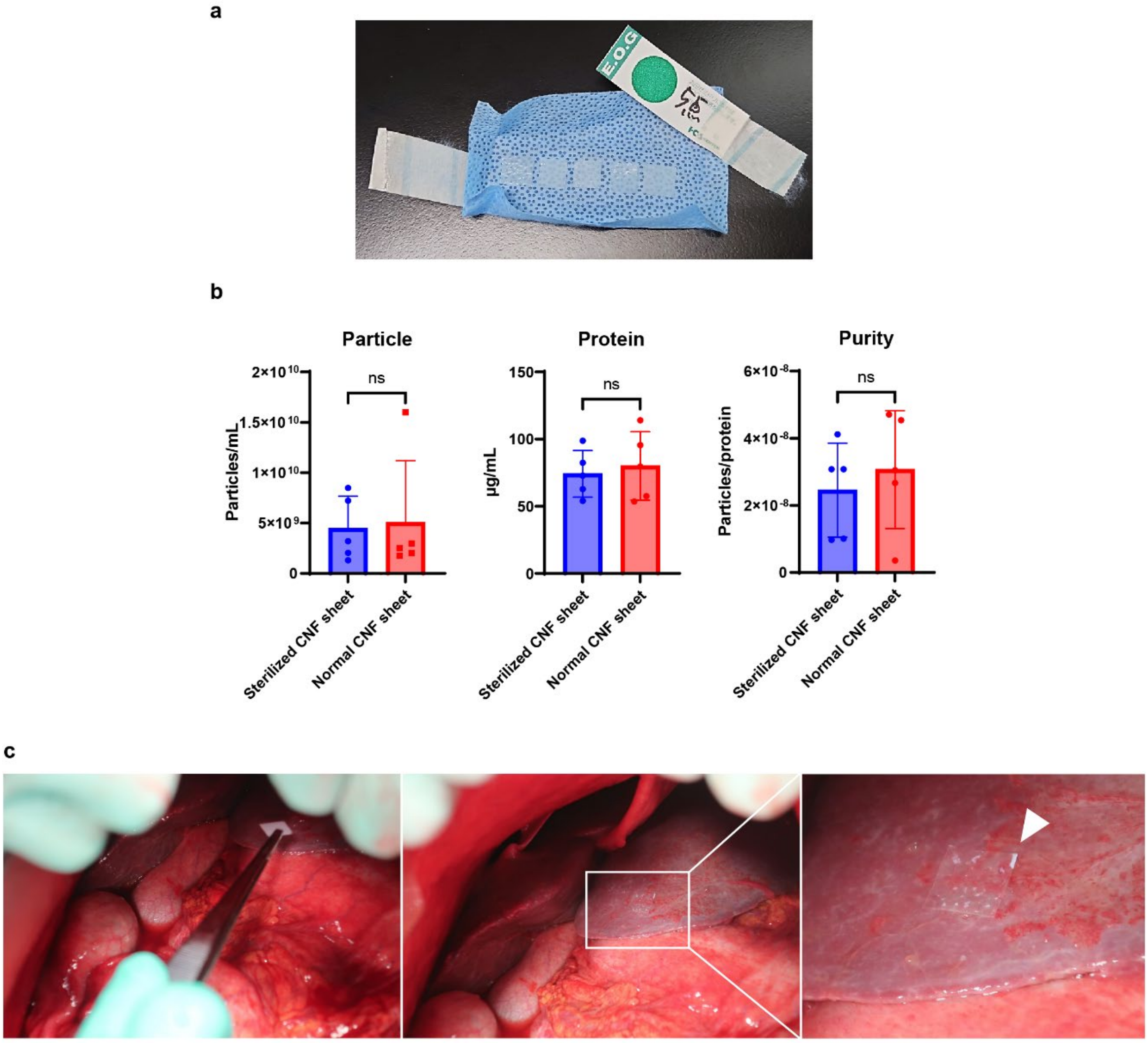

Supplementary Figure 10. (a) Representative photos of EOG-sterilized EV sheets for use during surgeries. (b) Quality check of EV sheet with/without EOG sterilization for EV capture performance. Particle numbers, protein concentrations and purities (particle number/protein concentration) were assessed. Data are presented as mean values with SD. The experiment was repeated at least three times. (c) Illustrative photos of EV sheets attached to liver surfaces.

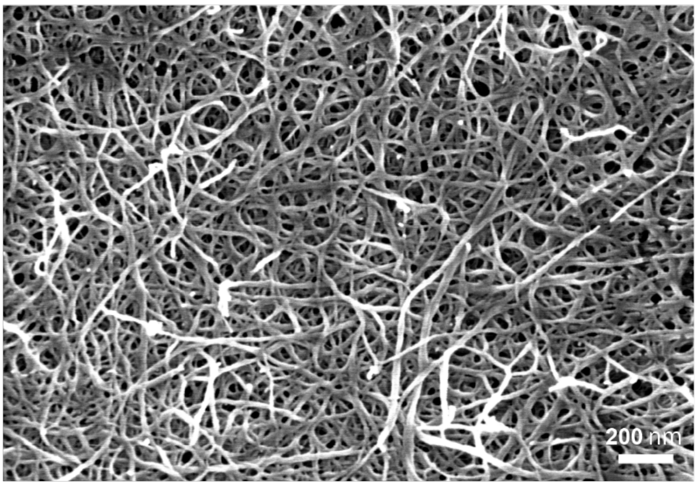

Supplementary Figure 11. FESEM image of post-sterilization CNF sheets.

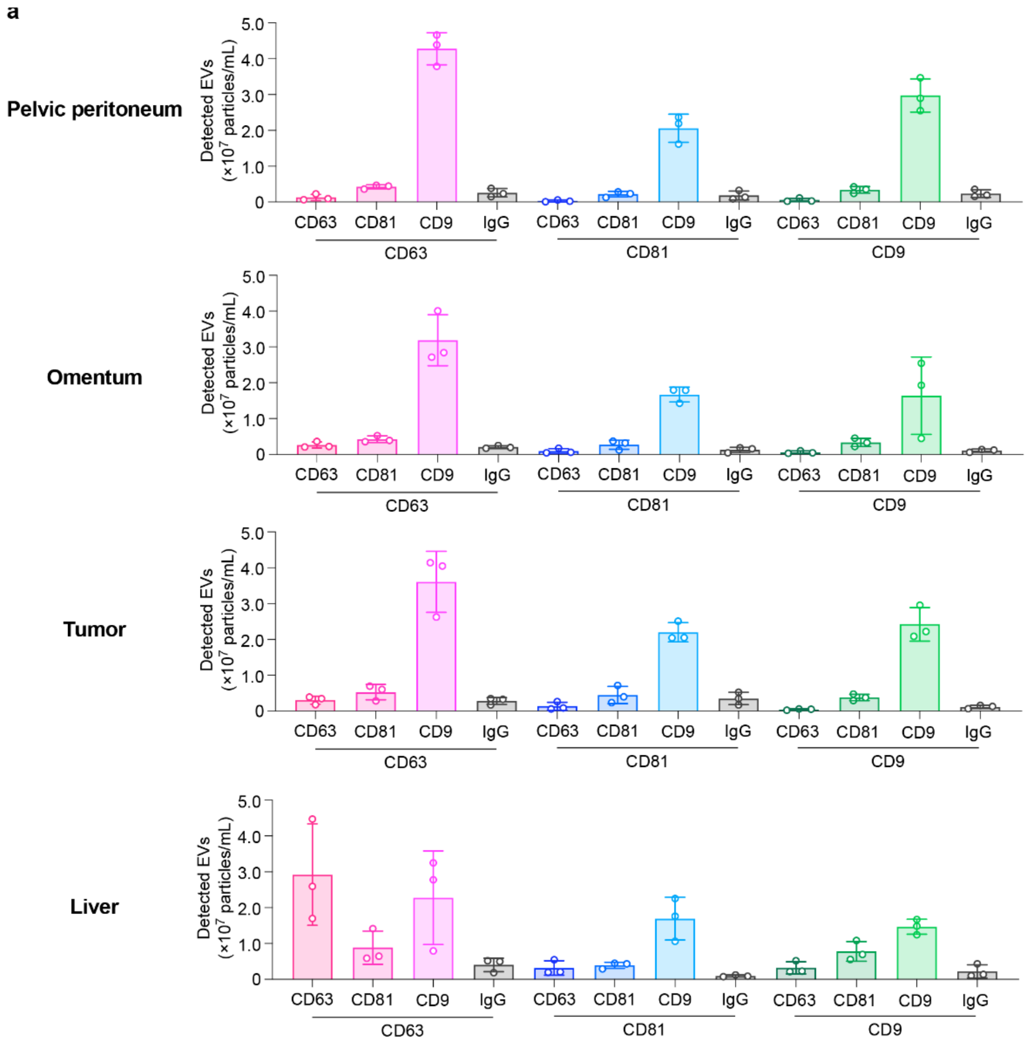

Supplementary Figure 12. EV marker proteins detected by Exoview of EVs recovered from EV-sheet EVs at the pelvic peritoneum, omentum, liver surface and tumour surface. Data are presented as mean values with SD. Each experiment was repeated at least three times.

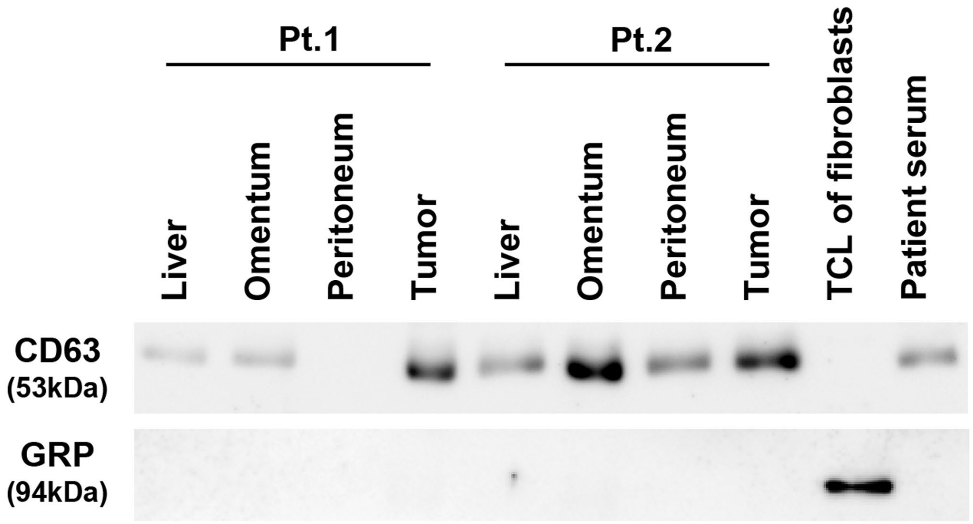

Supplementary Figure 13. Wester blotting analyses for EV and non-EV. “Fibroblasts” refer to the HFF2T cells, which are human foreskin derived fibroblasts immortalized with TERT protein markers. “Patient serum” refers to the serum EVs from patient no. 1 (**Supplementary Fig. 9a**) isolated by serial ultracentrifugation. TCL: total cell lysate. Each experiment was repeated at least three times.

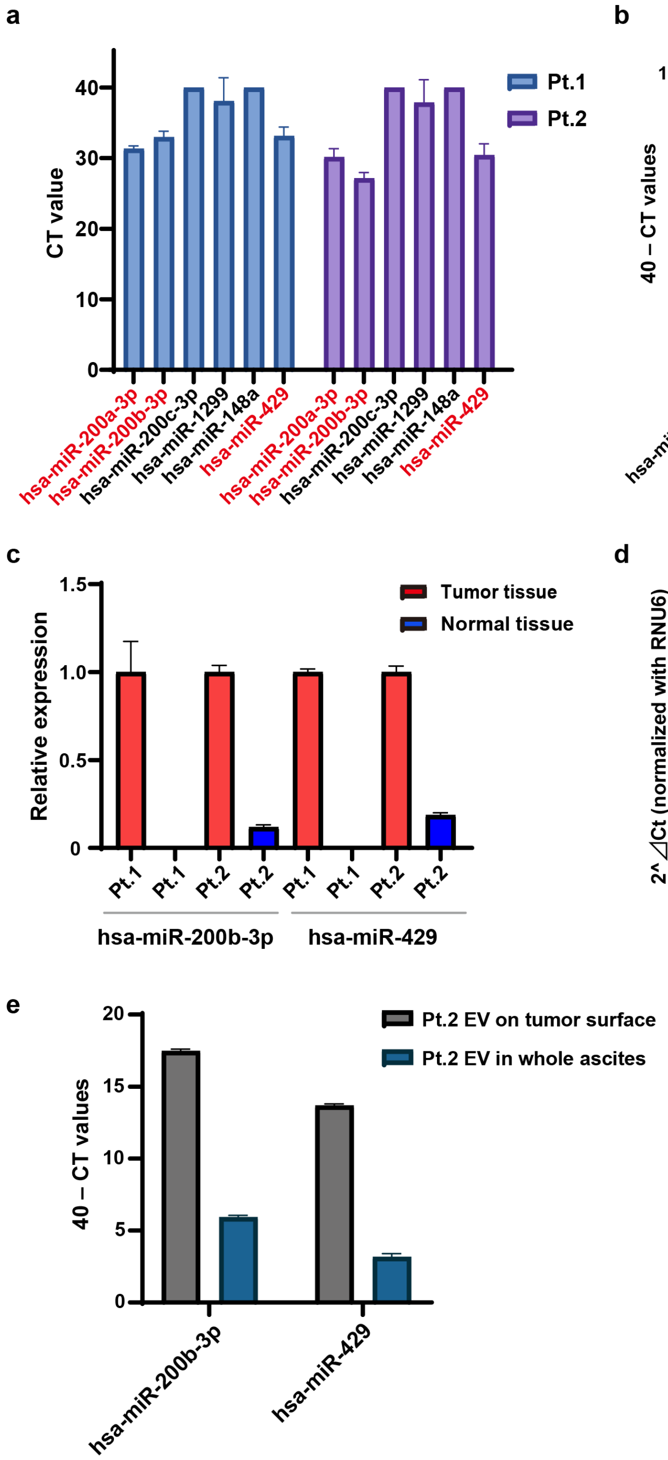

Supplementary Figure 14. qRT-PCR validation for EV-miRNAs. (a) CT values of hsa-miR-200a-3p, hsa-miR-200b-3p, hsa-miR-200c, hsa-miR-148a, hsa-miR-429, and hsa-miR-1299 in the serum EVs captured by CNF sheets. (b) CT values of hsa-miR-200a-3p, hsa-miR-200b-3p, and hsa-miR-429 in the serum EVs captured by CNF sheets. Data from pre- and post-surgery in the same patients were displayed. (c) Relative abundance of hsa-miR-200b-3p and hsa-miR-429 in tumor and normal tissues. Expression levels in tumor tissues were used as the reference and normalized to one. Normal tissues

were collected from the contralateral ovary without cancer. (d) Expression of hsa-miR-200b-3p and hsa-miR-429 in malignant tumor tissues from patients 1 and 2 and in a benign tumor confirmed to be cyst adenoma. (e) CT values of hsa-miR-200b-3p and hsa-miR-429 in ascites EVs from patient 2. All data are presented as mean values with SD. Each experiment was repeated at least three times.

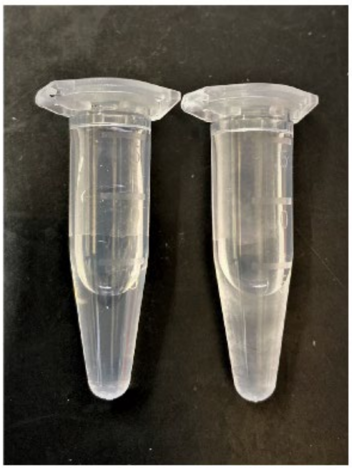

Supplementary Figure 15. The wet strength test of EV sheets (Lt) or normal pulp paper (Rt). The normal paper fibers unravel, and the solution becomes cloudy.

Supplementary Table. Cryo-EM data collection, refinement and validation statistics

	Images of EVs (Fig1f, 3e 5b)
**Data collection and processing**	
Magnification	30,000 (nominal magnification)
Voltage (kV)	200
Electron exposure (e–/Å^2)	40 - 50
Defocus range (μm)	-3.0 to -4.0
Pixel size (Å)	1.96
Symmetry imposed	n/a
Initial particle images (no.)	n/a
Final particle images (no.)	n/a
Map resolution (Å)	n/a
FSC threshold	
Map resolution range (Å)	n/a
**Refinement**	
Initial model used (PDB code)	n/a
Model resolution (Å)	n/a
FSC threshold	
Model resolution range (Å)	n/a
Map sharpening *B* factor (Å^2)	n/a
Model composition	n/a
Non-hydrogen atoms	
Protein residues	
Ligands	
B factors (Å^2)	n/a
Protein	
Ligand	
R.m.s. deviations	n/a
Bond lengths (Å)	
Bond angles (°)	
Validation	n/a
MolProbity score	
Clashscore	
Poor rotamers (%)	
Ramachandran plot	n/a
Favored (%)	
Allowed (%)	
Disallowed (%)	

All images in this study were captured as a snapshot, and any image analyses were not performed.
