# Supplementary material for: Spatial exosome analysis using cellulose nanofiber sheets reveals the location heterogeneity of extracellular vesicles
Source: Nat Commun. 2023 Nov 8;14:6915. doi: 10.1038/s41467-023-42593-9 (PMC10632339; doi:10.1038/s41467-023-42593-9)
Supplement: Supplementary file 3 — Description of Additional Supplementary Files [file 41467_2023_42593_MOESM3_ESM.pdf]

Description of Additional Supplementary Files

Supplementary Movies

Supplementary movie 1 The intact EV capture by CNF sheets. The EV capture and storage process using the CNF sheet consists of the following steps: EV capture by absorption of body fluid, EV storage by drying, washing, and EV release.

Supplementary movie 2 The extraction of EV-RNA from CNF sheets. After dropping 10 µL body fluids into the CNF sheets and drying and washing the CNF sheets, EV-RNAs were extracted by immersion in lysis buffer instead of recovery in PBS

Supplementary movie 3 CNF sheet attachment method in vivo. The EV capture process from moistened organs using the CNF sheet consists of the following steps: EV capture by absorption of ascites on organs, EV storage by drying, washing, and EV release.
