# Supplementary material for: Spatial exosome analysis using cellulose nanofiber sheets reveals the location heterogeneity of extracellular vesicles
Source: Nat Commun. 2023 Nov 8;14:6915. doi: 10.1038/s41467-023-42593-9 (PMC10632339; doi:10.1038/s41467-023-42593-9)
Supplement: Supplementary file 7 — Reporting Summary [file 41467_2023_42593_MOESM7_ESM.pdf]

nature portfolio

Corresponding author(s): Akira YOKOI, Takao YASUI

Last updated by author(s): October 6, 2023

Reporting Summary

Nature Portfolio wishes to improve the reproducibility of the work that we publish. This form provides structure for consistency and transparency in reporting. For further information on Nature Portfolio policies, see our Editorial Policies and the Editorial Policy Checklist.

Statistics

For all statistical analyses, confirm that the following items are present in the figure legend, table legend, main text, or Methods section.

n/a	Confirmed	
☐	☒	The exact sample size (*n*) for each experimental group/condition, given as a discrete number and unit of measurement
☐	☒	A statement on whether measurements were taken from distinct samples or whether the same sample was measured repeatedly
☐	☒	The statistical test(s) used AND whether they are one- or two-sided<br>*Only common tests should be described solely by name; describe more complex techniques in the Methods section.*
☐	☒	A description of all covariates tested
☐	☒	A description of any assumptions or corrections, such as tests of normality and adjustment for multiple comparisons
☐	☒	A full description of the statistical parameters including central tendency (e.g. means) or other basic estimates (e.g. regression coefficient) AND variation (e.g. standard deviation) or associated estimates of uncertainty (e.g. confidence intervals)
☐	☒	For null hypothesis testing, the test statistic (e.g. *F*, *t*, *r*) with confidence intervals, effect sizes, degrees of freedom and *P* value noted<br>*Give P values as exact values whenever suitable.*
☒	☐	For Bayesian analysis, information on the choice of priors and Markov chain Monte Carlo settings
☐	☒	For hierarchical and complex designs, identification of the appropriate level for tests and full reporting of outcomes
☐	☒	Estimates of effect sizes (e.g. Cohen's *d*, Pearson's *r*), indicating how they were calculated

Our web collection on statistics for biologists contains articles on many of the points above.

Software and code

Policy information about availability of computer code

Data collection	nanoparticle tracking analyzer: NanoSight NS300 (Malvern Panalytical Ltd., UK)<br>cryotransmission electron microscope (Terabase Inc., Okazaki, Japan)<br>ExoView R100 imager (NanoView Bioscience, Brighton, MA, USA)<br>small RNA sequencing: Illumina MiSeq or NextSeq (Illumina, San Diego, CA, USA)
Data analysis	small RNA sequencing analysis: CLC Genomics Workbench version 9.5.3 (Qiagen)<br>ExoScan 2.5.5 acquisition software (NanoView BIOSCIENCES, Brighton, MA, USA)<br>Statistical analyses were performed using RStudio (RStudio, Boston, MA, USA) and R software (ver. 4.0.3)

For manuscripts utilizing custom algorithms or software that are central to the research but not yet described in published literature, software must be made available to editors and reviewers. We strongly encourage code deposition in a community repository (e.g. GitHub). See the Nature Portfolio guidelines for submitting code & software for further information.

Data

Policy information about availability of data

All manuscripts must include a data availability statement. This statement should provide the following information, where applicable:

- Accession codes, unique identifiers, or web links for publicly available datasets
- A description of any restrictions on data availability
- For clinical datasets or third party data, please ensure that the statement adheres to our policy

The full small RNA expression profiles are stored in the Gene Expression Omnibus (GEO) database (GSE216745, GSE216793). The data were mapped to the miRbase 22 database (https://mirbase.org/). Other all data needed to evaluate the conclusions in the paper are presented in the paper and/or the Supplementary Materials. Additional data related to this paper may be requested from the authors.

Research involving human participants, their data, or biological material

Policy information about studies with human participants or human data. See also policy information about sex, gender (identity/presentation), and sexual orientation and race, ethnicity and racism.

Reporting on sex and gender	female
Reporting on race, ethnicity, or other socially relevant groupings	East Asian
Population characteristics	Between April 2020 and March 2022, serum and ascites samples of ovarian tumor patients were collected at Nagoya University Hospital (Nagoya, Japan), and tissue samples were also obtained under strict ethical approval by the Ethical Committee of Nagoya University Hospital, Japan. Six patients and age between 33 to 71.
Recruitment	Patients were recruited under written individual informed consent between April 2020 and March 2022. There is the only one bias whether the patient agree or not, but other selection bias did not exist.
Ethics oversight	the Ethics Committee of Nagoya University (approval number:2017-0053, 2021-0303)

Note that full information on the approval of the study protocol must also be provided in the manuscript.

Field-specific reporting

Please select the one below that is the best fit for your research. If you are not sure, read the appropriate sections before making your selection.

☒ Life sciences ☐ Behavioural & social sciences ☐ Ecological, evolutionary & environmental sciences

For a reference copy of the document with all sections, see nature.com/documents/nr-reporting-summary-flat.pdf

Life sciences study design

All studies must disclose on these points even when the disclosure is negative.

Sample size	The sample size used in this study was determined based on the expense of data collection, and the need to have sufficient statistical power.
Data exclusions	Data from Small RNA sequencing that did not meet the RPM criteria were eliminated.
Replication	For small RNA sequencing, samples were replicated in three times from the same spot. Due to the valuableness of human samples, all sample were collected in one time point at surgery. Regarding urine or saliva, post-surgery samples were also collected.
Randomization	No randomization is done in this study. This study showed the concept of the CNF sheet-quality, and not intended to clinical power. For this reason, randomization is not relevant to this study.
Blinding	To analyze molecular profiles of samples, background information is essential. This study was not to intend to show clinical effectiveness, and therefore blinding is not relevant to this study.

Reporting for specific materials, systems and methods

We require information from authors about some types of materials, experimental systems and methods used in many studies. Here, indicate whether each material, system or method listed is relevant to your study. If you are not sure if a list item applies to your research, read the appropriate section before selecting a response.

Materials & experimental systems

n/a	Involved in the study
☐	☒ Antibodies
☐	☒ Eukaryotic cell lines
☒	☐ Palaeontology and archaeology
☐	☒ Animals and other organisms
☐	☒ Clinical data
☒	☐ Dual use research of concern
☒	☐ Plants

Methods

n/a	Involved in the study
☒	☐ ChIP-seq
☒	☐ Flow cytometry
☒	☐ MRI-based neuroimaging

Antibodies

Antibodies used

ExoView® Tetraspanin Kits (NanoView Bioscience, Brighton, MA. Ca No. N/A) contained anti-CD9 (clone H19a), anti-CD63 (clone H5C6) and anti-CD81 (clone JS81).
For western blottings, primary Ab: rabbit monoclonal anti-CD63 (EXOAB-CD63A-1; System Biosciences, LLC, CA, USA; dilution 1:1,000) and mouse monoclonal anti-GRP (sc-393402; Santa Cruz Biotechnology; dilution 1:100) and secondary Ab: HRP-conjugated mouse anti-rabbit IgG (NA934-1ML; Cytiva Lifesciences, USA; dilution 1:5,000) or anti-mouse IgG (NA931-1ML; Cytiva; dilution 1:2,000)

Validation

Regarding ExoView® system, all antibodies were validated by NanoView Bioscience (Brighton, MA, USA)
For western blottings, each Ab was validated in each venders.

Eukaryotic cell lines

Policy information about cell lines and Sex and Gender in Research

Cell line source(s)

ID8 cells, a murine epithelial ovarian cancer cell line established from C57BL/6 murine ovarian surface epithelial cells which is described previously (doi:10.3892/ijo.2019.4845).

Authentication

Dr Katherine Roby (University of Kansas Medical Center) authenticated the cells.

Mycoplasma contamination

routinely tested as negative for mycoplasma infection

Commonly misidentified lines
(See ICLAC register)

N/A

Animals and other research organisms

Policy information about studies involving animals; ARRIVE guidelines recommended for reporting animal research, and Sex and Gender in Research

Laboratory animals

Eight-week-old female BALB/cSlc-nu/nu mice were purchased from Japan SLC, Inc. (Shizuoka, Japan). All mice were housed in an animal facility at Nagoya University School of medicine under the condition of standard 12h/12h light/dark cycles with food, water, and diets. The rooms were 18-24 degrees celsius and around 50% humidity.

Wild animals

No wild animals were used in the study.

Reporting on sex

female

Field-collected samples

No field collected samples were used in the study.

Ethics oversight

Experiments were supervised and approved by the Center for Animal Research and Education (CARE) at Nagoya University (approval number M220393-004)

Note that full information on the approval of the study protocol must also be provided in the manuscript.

Clinical data

Policy information about clinical studies
All manuscripts should comply with the ICMJE guidelines for publication of clinical research and a completed CONSORT checklist must be included with all submissions.

Clinical trial registration

Clinical data in this study is categorized in observational/low-interventional clinical trial.

Study protocol

Clinical data in this study is categorized in observational/low-interventional clinical trial.

Data collection

Clinical information and biospecimens were collected perioperatively.

Outcomes	Evaluated by small RNA sequence data.
